# Clinically Contextualized State Spaces Provide Continuous Representations of Sepsis

**DOI:** 10.21203/rs.3.rs-11221040/v1

**Published:** 2026-10-05

**Authors:** Seibi Kobara, Morgan Kay, Tilendra Choudhary, Vijay Krishnamoorthy, Mihai V. Podgoreanu, Yoshiro Hayashi, Suresh M. Agarwal, Rishikesan Kamaleswaran

**Affiliations:** Duke University School of Medicine; Duke University; Duke University School of Medicine; Duke University School of Medicine; Duke University School of Medicine; Kameda Medical Center; Duke University School of Medicine; Duke University School of Medicine

**Keywords:** Representation learning, foundation model, endotype discovery, critical illness, sepsis

## Abstract

Precision medicine increasingly leverages molecular and clinical similarity to identify patient subgroups and guide individualized care. However, conventional cross-sectional approaches often characterize biological states independently of the clinical context in which they arise. Similar laboratory profiles may have different clinical implications depending on subsequent trajectory, while biologically distinct states may share similar clinical outcomes. Consequently, clustering based solely on clinical trajectory-agnostic clustering may incompletely capture clinically relevant heterogeneity.

To address these limitations, we introduce CERL (Contextual Endotype and State Representation Learning), a clinical trajectory-informed representation learning framework that integrates clinical contexts to construct patient-state representations from clinical data. Rather than defining patients solely through discrete clusters, CERL learns a continuous clinical state space that preserves biological heterogeneity while organizing states according to clinically relevant trajectories.

Across both simulated and real datasets, CERL robustly identified latent patient groups and reliably reproduced the learned patient groups in validation. In a proof-of-concept application across multi-institutional critical care cohorts, CERL identified three reproducible clinical state groups embedded within a state representation. The representation captured systematic variation in routine laboratory phenotypes and demonstrated clinical trajectory-specific redistribution dynamics within the continuous state space.

These findings establish CERL as a framework for generating clinically contextualized state representations that integrate biological characteristics with clinical trajectories, providing a foundation for longitudinal pathological progression modeling and future digital twin approaches in critical care.

## Introduction

Precision medicine in sepsis remains an unmet need in critical care, given its unacceptably high mortality despite advances in supportive care^1–3^. Beyond its high mortality, sepsis is associated with prolonged sequelae that may persist for over a decade, including physical impairment, ventilator dependence, diminished quality of life, increased caregiver burden, and substantial societal costs related to healthcare utilization^4–9^. These diverse consequences highlight the need for a holistic approach to sepsis that extends beyond survival alone^10,11^.

Sepsis represents a heterogeneous syndrome rather than a single disease entity, arising from diverse infectious insults and host responses that result in substantial variation in organ dysfunction, immune response, treatment effects, and clinical outcomes^3,12^. Furthermore, clinical states are not static but evolve dynamically throughout the course of sepsis, and patients with similar presentations may subsequently follow divergent clinical trajectories^13^.

Recent advances in translational and computational research have sought to address this heterogeneity through biological endotyping and data-driven clinical phenotyping. Using bulk transcriptomic data, Sciclula et al. identified four molecular response endotypes^14^. Sweeney et al. identified three endotypes in bacterial sepsis^15^. More recently, single-cell RNA-sequencing has enabled high-resolution characterization of immune heterogeneity and continuoue immune severity states across viral and bacterial sepsis^16^. Moore et al. collectively characterized four endotypes spanning detrimental myeloid cluster, protective myeloid cluster, protective lymhoid cluster, and mixed myloid or lymphoid cluster with coagulopathy^17^. In parallel, large clinical cohorts have enabled identification of clinical phenotypes using dimensionality reduction and unsupervised machine learning approaches^12,13,18,19^. A large-language model was applied to heterogenous clinical data to generate contextual representations, which were subsequently used to identify latent patient groups with distinct phenotypic characteristics^18^. Reflecting the temporal variability of the clinical states, dynamic time warping has been used to characterize temporal patterns of organ dysfyunction^13^.

Despite these progressive advancements in subgroup analyses, a fundamental representation gap remains. Cross-sectional endotyping primarily organizes patients according to biological or physiological similarity; yet biological similarity does not necessarily imply similarity in subsequent clinical trajectory. Conversely, biologically distinct states may be associated with similar downstream outcomes. Longitudinal clustering approaches capture temporal patterns of state evolution. However, these patterns are typically discovered in an outcome-agnostic manner. Consequently, the trajectories identified from the biological or physiological data are not necessarily those most informative of clinically meaningful outcomes. Supervised dimentionality-reduction approaches can incorporate outcome information into dimension-reduced space, such as Partial Least Squares Discriminant Analysis (PLS-DA)^20,21^, but are commonly defined with a single outcome. Sepsis, however, is characterized by multiple interrelated clinical trajectories, in which patients can recover early with or without mechanical ventilation or mortality outcome with or without renal replacement therapy.

Here, we present Contextual Endotype and State Representation Learning (CERL), a new framework to provide clinically contextualized, continuous clinical-state representations, leveraging Tabular Prior-data Fitted Network (TabPFN)^22^. By incorporating multiple outcome context during representation learning, CERL extends conventional phenotype discovery beyond biological similarity alone and reduces reliance on post-hoc associations between discovered subgroups and clinical outcomes. CERL (1) generates continuous clinical-state representation embeddings; (2) organizes clinically relevant variation within similar biological states; (3) enables discrete state groups to be derived using conventional unsupervised learning; and (4) provides a continuous manifold clinical state space that may serve as a foundation for future temporal pathological modeling, causal modeling, and digital twin construction. Although alternative representation-learning architectures, including supervised autoencoders, multitask neural networks could be used to construct similar embeddings, CERL is defined by the clinical-state formulation rather than by a specific architecture. The central contribution is the construction of a clinically contextualized state space in which clinical trajectories contribute to the organization of physiologic states, enabling subsequent analysis of state transitions and pathological progression dynamics.

We first systematically evaluated CERL using synthetic data with predefined biological states and clinical outcomes. For real-world evaluation, we then applied CERL to a large multicenter cohort of patients meeting Sepsis-3 criteria^23^ within the eICU Collaborative Research Database^24^ to characterize clinical-state organization across divergent patient clinical trajectories.

## Methods

### Contextual endotype and state representation learning framework

We developed Contextual Endotype and State Representation Learning (CERL), a representation-learning framework that derives latent endotypes and clinical state representations using multiple clinical outcomes as contextual anchors (Figure 1). CERL leverages TabPFN (ver. 8.4.0)^22^, a foundation model pretrained on over 100 million synthetic datasets, as its contextual representation-learning backbone.

Given an input feature matrix and a set of user-defined clinical outcomes, CERL learns a nonlinear latent representation of the input features conditioned on outcome context. Unlike conventional outcome-informed dimensionality-reduction approaches, such as PLS-DA^20,21^, CERL can incorporate multiple, partially overlapping clinical outcomes simultaneously. For example, in critical care, these outcomes may include mortality, invasive mechanical ventilation, and renal replacement therapy.

The resulting CERL representation preserves variation in the input feature space while incorporating information about its nonlinear associations with multiple clinical trajectories. Thus, observations with similar measured biological profiles are not required to occupy identical positions in the latent representation when their associated clinical contexts differ. Conversely, distinct biological profiles may occupy related regions of the representation when they share clinically relevant context. CERL therefore produces a continuous, outcome-informed state space rather than directly assigning observations to predefined discrete endotypes. Discrete clinical-state groups can subsequently be derived from the CERL representation using unsupervised clustering. Detailed methodology is provided in the Supplementary Methods.

### Synthetic data experiments

We conducted synthetic data experiments to evaluate whether CERL could recover predefined endotypes under not only controlled linear but also nonlinear data-generating mechanisms. Simulated datasets comprised 20 correlated continuous features, from which ground-truth endotypes were defined through an underlying continuous function of the input features. Multiple binary clinical outcomes were subsequently generated with prespecified endotype-dependent probabilities. We evaluated sample sizes of 1,000, 1,500, and 3,000, with two to four latent states. In each scenario, 50 independent simulation replicates were constructed. Detailed data-generating procedures are provided in the Supplementary Methods.

Recovery of the predefined endotypes was quantified using the adjusted Rand index (ARI), and the recovery using CERL was compared with other conventional unsupervised approaches, including K-means clustering, Bayesian Gaussian mixture (BGM) modeling, agglomerative clustering, and HDBSCAN, as well as PLS-DA^20,21^-based outcome-informed dimensionality reduction followed by clustering.

Generalizability of the CERL representation was additionally evaluated in a held-out test set comprising 33% of observations. CERL representations for the test set were generated using only the development-set features and outcomes as contextual information, and K-means clustering was fitted exclusively on the development-set representations before being applied to the held-out test set.

### Reproducibility assessment of CERL using critical care cohort data

We used the eICU Collaborative Research Database (eICU-CRD)^24^, which contains 200,859 ICU encounters from 139,367 unique patients across multiple U.S. hospitals. Patients who met Sepsis-3 criteria^23^ were included in the analysis, resulting in 2,127 unique ICU encounters and patients (Supplementary Figure S1). Detailed daily SOFA reconstruction and procedures used to retrospectively identify sepsis condition are provided in Supplementary information. We further identified clinical trajectories according to the reconstructed daily SOFA score trajectories. Following the K-means clustering, we manually grouped patients into four groups: acute recovery, late recovery, acute death, and late death (Supplementary Figure S2). The detailed methodology is provided in the Supplementary Methods. Briefly, these clusters are characterized as follows:

- Acute recovery: SOFA scores rapidly declined to < 2 within three days after ICU admission.
- Late recovery: Persistently high SOFA scores and gradually declining by 7 to 20 days after ICU admission.
- Acute death: Rapid progression to death, within the first 10 days after ICU admission.
- Late death: Later death occurring between 10 and 21 days after ICU admission.

To assess the reproducibility of the representation embeddings and biological consistency of CERL, we conducted two institution-held-out validation experiments. Within the eICU cohorts, we split into a derivation cohort and validation cohort using institution labels, rather than using patient-level random splitting. This approach reduces the risk of data leakage arising from institution-specific practice patterns (e.g., laboratory testing and treatment practices) and institution-specific patient characteristics. Detailed procedures for these validation experiments are provided in Supplementary information.

For each validation experiment, CERL representation was constructed using the derivation cohort, and K-means clustering was fitted to the resulting representation to define three discrete CERL states (S0, S1, and S2). The fitted CERL representation model and the trained K-means centroids were subsequently applied to observations in the validation cohort to generate CERL embeddings and CERL states.

Reproducibility was evaluated at three levels. First, state reproducibility of the validation representation embeddings were compared with the derivation embeddings using cosine similarity. Second, biological reproducibility was assessed using laboratory marker distribution for each derived states, using Pearson correlation. Lastly, clinical reproducibility was evaluated by estimating the proportion of each prespecified clinical trajectory within each CERL state. Mean absolute error (MAE) across six clinical trajectories and states was computed.

Because individual patients contributed multiple longitudinal observations, patient-level block bootstrap resampling was used to estimate uncertainty while preserving within-patient dependence. Reproducibility statistics and state-specific outcome proportions were recalculated for each bootstrap replicate, and 95% confidence intervals were obtained from the resulting bootstrap distributions.

### Benchmarking with conventional unsupervised machine learning models

CERL was designed as a biological and clinical state representation framework in which multiple, potentially overlapping clinical trajectories provide contextual information during representation learning. To evaluate whether this contextual representation provides additional information beyond conventional outcome-agnostic phenotyping, we benchmarked CERL against K-means clustering and a BGM applied directly to the input laboratory measurements. Comparisons focused on three complementary properties: state reproducibility, biological reproducibility, and clinical trajectory informativeness. A detailed procedure is provided in the Supplementary Methods. In particular, clinical trajectory informative evaluation was designed to evaluated whether the derived state partitions retained information associated with each of the six prespecified clinical trajectories. Clinical trajectory discrimination was quantified using the area under the receiver operating characteristic curve (AUROC) and area under the precision-recall curve (AUPRC). The reason for selecting these metrics over other classification metrics, such as accuracy or F1 score, is provided in the Supplementary Methods. Patient-level block bootstrap resampling was used to derive 95% CIs for the mean of metrics.

### A multi-institution sepsis database application

We applied CERL to the full sepsis cohort within the eICU-CRD^24^ to demonstrate its real-world application. The resulting high-dimensional CERL representations were visualized using Potential of Heat-diffusion for Affinity-based Transition Embedding (PHATE)^25^. PHATE constructs diffusion-based distances from local affinities, allowing local relationships to be integrated into the broader manifold structure. We selected PHATE because this property facilitates visualization of continuous and potentially transitional organization within the CERL representation space while preserving local relationships among observations. To improve state interpretation, we used K-means clustering onto the derived representation embeddings and described the proportion of six clinical trajectories and lab values used for the input of CERL.

To evaluate whether CERL introduced clinical trajectory-related organization beyond that captured by conventional outcome-agnostic phenotyping, we applied K-means clustering directly to the laboratory measurements used as inputs to CERL. Within each laboratory-defined cluster, we examined the organization of observations in the continuous CERL representation and calculated pairwise distances between observations belonging to the same versus different clinical trajectories. This analysis assessed whether CERL captured additional trajectory-related organization among observations with otherwise similar laboratory-defined biological states.

All machine-learning analyses were performed in Python (ver. 3.11). Unsupervised machine learning methods were implemented using scikit-learn (ver. 1.9.0)^26^. Statistical analyses and figure generation were performed using R (version 4.5.3).

## Results

### CERL reliably recovered predefined endotypes under nonlinear endotype generation mechanisms

We developed a contextual endotype and state representation learning (CERL) leveraging TabPFN^22^, a foundation model pretrained on over 100 million synthetic datasets and natively robust to noise and missingness. CERL was developed to address a fundamental challenge in biologic endotype discovery: clinically meaningful endotypes may arise from complex interactions among laboratory measurements and may not be identifiable through conventional clustering approaches. Most existing endotype discovery methods rely on fixed distance metrics or distribution-based unsupervised clustering. While these approaches can separate patients according to dominant biological signals, such as immune activation or organ dysfunction, they do not explicitly ensure that identified groups correspond to clinical trajectories associated with recovery or mortality. Consequently, patients with similar laboratory measurements at a given time point may be assigned to the same cluster despite markedly different clinical trajectories. CERL addresses this limitation by learning contextual representations that preserve nonlinear relationships among routine laboratory measurements while organizing patients according to shared clinical trajectories. Unlike conventional endotyping approaches that rely on feature similarity or a single outcome, CERL is trained using multiple trajectory-derived outcome labels, enabling the learned representations to capture latent physiologic states associated with diverse patterns of recovery and deterioration.

To evaluate whether CERL improved recovery of latent endotype structure, we conducted systematic simulation experiments across varying sample sizes, number of simulated endotypes, and degree of nonlinear associations between observed features and latent endotypes. Under each simulation setting, we generated synthetic datasets. We then constructed endotype assignments based on the simulated data and downstream outcome labels conditioned on endotype status. These simulations were designed to mimic settings in which clinically relevant latent states influence both observed measurements and future outcomes but are not directly observable.

We benchmarked CERL against conventional unsupervised clustering approaches using the Adjusted Rand Index (ARI), which quantifies agreement between inferred and reference cluster assignments while accounting for chance agreement (ARI = 0 indicates random agreement and ARI = 1 indicates perfect agreement). ARI was used to assess the ability of CERL to recover predefined latent endotype assignments.

Across simulation settings, CERL consistently outperformed conventional K-means clustering applied directly to the original feature space (Figure 2 and Supplementary Figure S3). With a sample size of 1500 subjects, three endotypes, and a nonlinear endotype-generation mechanism, CERL achieved a mean ARI of 0.539 (95% CI: 0.509, 0.570), whereas conventional K-means achieved a mean ARI of 0.058 (95% CI: 0.047, 0.072).

### CERL showed robust reproducibility using critical care cohort data

To evaluate the representation-state reproducibility, we used a multicenter cohort of patients meeting Sepsis-3 criteria^23^ within the eICU-CRD^24^. Reproducibility was evaluated across three complementary dimensions: representation-space reproducibility, biological reproducibility, and clinical trajectory reproducibility.

First, state reproducibility was evaluated using cosine similarity between state-specific mean CERL representation embeddings in the derivation and validation cohorts. In the first validation experiment, cosine similarities between corresponding states ranged from 0.894 to 0.974, whereas similarities between non-corresponding states ranged from −0.535 to −0.397. Similarly, in the second validation experiment, cosine similarities between corresponding states ranged from 0.888 to 0.943, while similarities between non-corresponding states ranged from −0.624 to −0.280 (Supplementary Figure S4-A).

Second, we evaluated biological reproducibility by comparing state-specific mean laboratory profiles between the derivation and validation cohorts. Pearson correlation between corresponding state-specific laboratory profiles ranged from 0.929 to 0.983 in the first validation experiment and from 0.852 to 0.944 in the second validation experiment (Supplementary Figure S4-B).

Finally, clinical reproducibility was assessed by comparing the state-specific proportions of the six clinical trajectories between the derivation and validation cohorts. Across most state-trajectory combinations, the clinical trajectory proportions observed in the derivation cohort fell within the 95% CIs of the corresponding proportions estimated in the validation cohorts (Supplementary Figure S4-C). The MAE was 0.075 in the first validation experiment and 0.051 in the second validation experiment.

### Clinical trajectory information in CERL-derived states compared with conventional unsupervised phenotyping

CERL was designed as a biological and clinical state representation framework in which multiple, potentially overlapping clinical trajectories provide contextual information during representation learning. To evaluate whether this contextual representation provides additional information beyond conventional outcome-agnostic phenotyping, we benchmarked CERL against K-means clustering and a Bayesian Gaussian mixture model (BGM) applied directly to the input laboratory measurements using three complementary metrics: biological reproducibility, clinical reproducibility, and clinical trajectory informativeness.

First, biological reproducibility was evaluated by comparing state-specific mean laboratory profiles between the derivation and validation cohorts. Using K-means, Pearson correlations between corresponding states ranged from 0.958 to 0.982 in the first validation experiment and 0.921 to 0.964 in the second validation experiment (Supplementary Figure S5-A). In contrast, BGM showed lower correlations between corresponding states (Supplementary Figure S5-A).

Second, clinical reproducibility was assessed by comparing the state-specific proportions of the six clinical trajectories between the derivation and validation cohorts. Both K-means and BGM showed broadly preserved clinical trajectory distributions for each state. The MAE values between derivation and validation cohorts were 0.071 and 0.047 for K-means in the first and second validation experiments, respectively, and 0.079 and 0.057 for BGM (Supplementary Figure S5-B).

Finally, we evaluated the extent to which the derived state partitions retained information associated with the six clinical trajectories using AUROC and AUPRC. Although states derived using conventional K-means and BGM already retained substantial clinical trajectory information, CERL showed greater AUPRC for acute recovery, late recovery, and invasive mechanical ventilation (Supplementary Figure S6).

### Contextual clinical state representation using a large-scale real-world dataset

We applied CERL to the full sepsis cohort of eICU-CRD^24^ to demonstrate its application to longitudinal real-world clinical data. CERL representations derived from longitudinal laboratory measurements were projected into a continuous clinical-state space using PHATE, and three discrete CERL states (S0, S1 and S2) were derived by applying K-means clustering to the CERL representations (Figure 3-A).

The resulting CERL states showed distinct clinical trajectory profiles. S0 was relatively enriched for acute death and invasive mechanical ventilation without renal replacement therapy, whereas S1 was characterized by acute recovery. S2 showed greater representation of renal replacement therapy, and this state-specific enrichment persisted across the longitudinal observation period (Figure 3-A, -B, and -C and Supplementary Figure S7 and S8).

For other clinical information, including days relative to ICU admission, medical or surgical admission type, or sepsis shock state, occupied preferential but overlapping regions with CERL states on the continuous CERL manifold (Supplementary Figure S7).

CERL states were also characterized by distinct biological profiles. S1 and S2 showed relatively lower white blood cell counts, whereas S2 was characterized by relative thrombocytopenia and higher blood urea nitrogen and creatinine concentrations (Figure 3-D, Supplementary Figure S9). The biological characteristics were also preserved longitudinally. State-specific laboratory profiles remained distinct across multiple days surrounding ICU admission, with particularly persistent differences in hematologic and renal markers (Supplementary Figure S10). S1 and S2 showed persistently lower WBC and hematocrit concentrations than S0, while S2 showed persistent thrombocytopenia remaining below 150 kmcL. Notably, S2 also showed persistently higher BUN and creatinine concentrations over time.

The continuous CERL representation further showed outcome-specific temporal redistribution within the clinical-state space. Observations associated with acute recovery became progressively concentrated within the left region of the PHATE manifold overtime, with a similar directional pattern observed for late recovery. In contrast, observations associated with acute and late death remained distributed across a broader region of the manifold and showed temporal redistribution toward the right region of the representation space (Figure 3-E and Supplementary Figure S11). Mapping individual laboratory measurements onto the PHATE manifold further showed spatially organized, continuous variation in biological features (Supplementary Figure S12).

We finally evaluated whether CERL introduced clinical trajectory-related organization beyond that captured by conventional outcome-agnostic phenotyping. K-means clustering was applied directly to the laboratory measurements, with K=4 selected based on the silhouette score, yielding four laboratory-defined states (Lab-C-0 to Lab-C-3). These states were visualized in both the laboratory-derived and CERL-derived PHATE representations (Supplementary Figure S13-A and B). Laboratory-defined states showed substantial overlap with CERL states. We subsequently compared pairwise distances between observations belonging to the same versus different clinical trajectories within each laboratory-defined state. The ratio of between-trajectory to within-trajectory distances was greater in the CERL representation than in the laboratory-derived representation (1.164 vs. 1.061) (Supplementary Figure S13-C).

## Discussion

Clinical trajectory-informed latent representation learning can identify not only clinical and biological states but also states contextualized by multiple clinical trajectories simultaneously. We developed CERL, a framework for generating such trajectory-aware representations, and demonstrated its fidelity in recovering predefined endotypes in synthetic experiments. When applied to the eICU-CRD^24^, CERL generated a continuous representation space that captured clinically and biologically relevant variation across divergent clinical trajectories. Compared with conventional unsupervised phenotyping approaches, CERL-derived states retained greater or comparable outcome-relevant information while preserving a continuous representation that can support subsequent longitudinal analyses and, potentially, future digital-twin modeling.

An important feature of CERL is that clinical context is incorporated during representation learning rather than evaluated only after phenotypes have been identified. Most existing endotype-discovery approaches in critical care^12–19^ rely on unsupervised methods that organize observations according to similarity in the measured feature space. Although these approaches have identified reproducible and clinically important phenotypes, the structures they discover are not explicitly conditioned on subsequent clinical trajectories. Consequently, biologically similar states may be grouped together despite their association with divergent clinical courses, while biologically distinct states associated with similar trajectories may remain separated. CERL addresses this distinction by using multiple clinical outcomes as contextual information, allowing nonlinear relationships between the observed biological state and the broader clinical course to contribute to the learned representation. Furthermore, CERL provides a continuous representation of clinical states. As demonstrated in the sepsis cohorts, the resulting state space provides a framework for investigating how patients transition between physiologic states over time and for identifying factors associated with those transitions. Together, CERL should be viewed not simply as a phenotyping framework, but as a foundation for future data-driven simulation and digital twin approaches that integrate clinically contextualized state representations with temporal disease dynamics.

Several limitations warrant consideration. First, the real-world application included repeated observations from individual patients. CERL generates a representation for each observation but does not, by itself, model the within-patient dependence among longitudinal observations. Consequently, downstream statistical analyses of CERL representations should explicitly account for repeated measurements and within-patient correlation using appropriate longitudinal approaches, such as mixed-effects or other repeated-measures models. Similarly, movement within the CERL representation space should not necessarily be interpreted as a smooth biological trajectory without an explicit longitudinal model.

Second, CERL representations are conditional on the clinical outcomes specified during representation learning. Outcomes must therefore be defined a priori, and the resulting representation reflects the prespecified clinical context encoded by those outcomes. Addition of a new outcome requires reconstruction of the contextual representation rather than retrospective interpretation of the existing embedding as if that outcome had originally informed it. Careful outcome selection based on clinical rationale and, where appropriate, expert consensus is therefore important. This dependence should be viewed as both a limitation and a defining property of CERL: unlike outcome-agnostic phenotyping, the intended clinical context of the representation is explicitly specified.

Third, the real-world evaluation was performed within a single critical-care database. Although the eICU Collaborative Research Database includes patients from multiple institutions, heterogeneity in laboratory measurement and clinical practice across healthcare systems may influence the learned representation. Explicit modeling of provider- and institution-level heterogeneity represents an important direction for future applications.

## Conclusion

CERL provides a framework for learning continuous clinical-state representations that integrate biological and physiological similarity with context from multiple clinical trajectories. By incorporating clinical outcomes during representation learning rather than evaluating their relevance exclusively after phenotype discovery, CERL provides a complementary approach to conventional outcome-agnostic endotyping. In sepsis, CERL identified continuous and discrete clinical-state organization associated with divergent patient trajectories using routinely collected clinical data. Further validation across independent populations and data modalities may establish CERL as a generalizable representation framework for precision phenotyping and future longitudinal and digital-twin modeling.

## Supplementary Material

Supplementary Files

This is a list of supplementary files associated with this preprint. Click to download.

- CERLsupplement.pdf

## Figures and Tables

**Figure 1:**
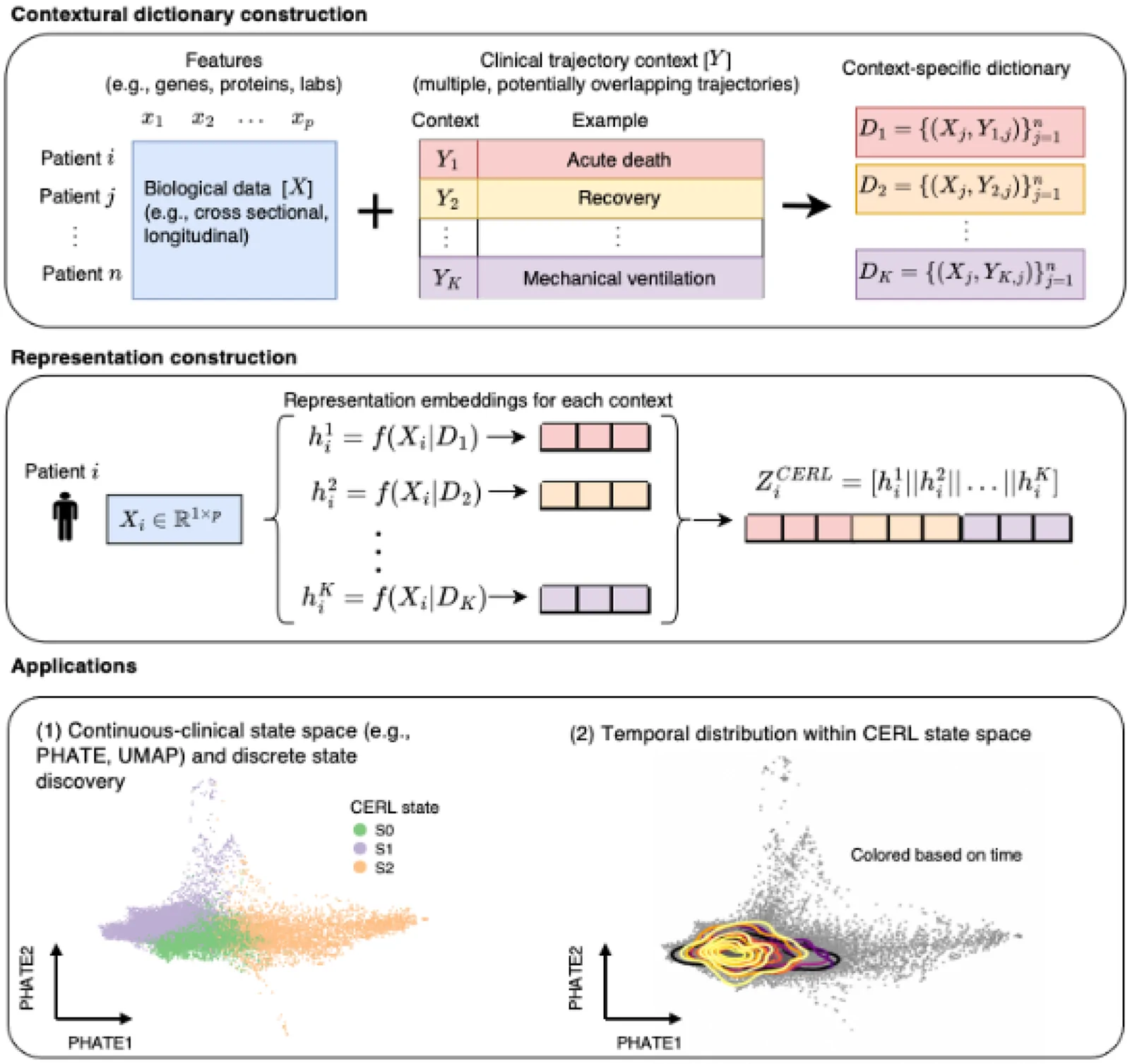
Overview of the Contextual Endotype and State Representation Learning (CERL). CERL constructs continuous biological and clinical-state representations by contextualizing input features with multiple, potentially overlapping clinical trajectories. For each clinical trajectory, input features and corresponding trajectory information define a context-specific dictionary that provides the reference context for representation learning. For a query observation, CERL generates a trajectory-specific representation conditional on each dictionary, and the resulting representations are concatenated to form a unified CERL representation. The continuous CERL state space can subsequently be interrogated using dimensionality-reduction approaches such as PHATE, discretized into state groups when categorical phenotypes are desired, and used to characterize temporal redistribution of clinical populations across the representation space.

**Figure 2:**
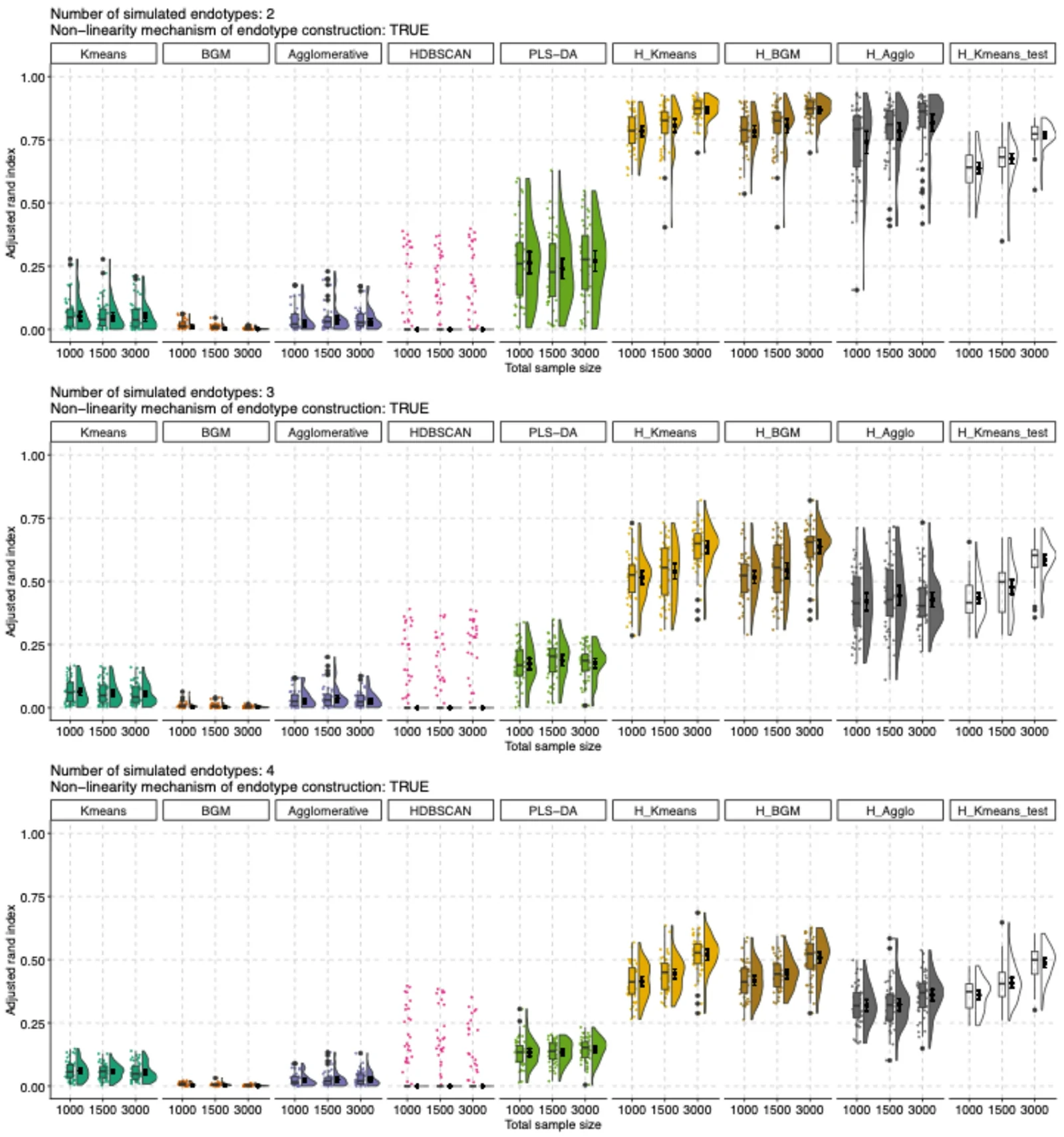
Synthetic data evaluation of CERL for latent endotype recovery. Synthetic datasets were generated from correlated multivariate normal distributions, and predefined latent endotypes were constructed using nonlinear functions of the simulated features. Multiple partially overlapping clinical outcomes were subsequently generated conditional on the predefined endotypes and used as clinical trajectory context for CERL. Data were separated into development and held-out validation sets, with validation representations generated using only the development-set context. Recovery of the predefined ground-truth endotypes was quantified using the adjusted Rand index (ARI). CERL was compared with conventional unsupervised clustering approaches and outcome-informed dimensionality reduction across varying sample sizes and numbers of predefined endotypes.

**Figure 3:**
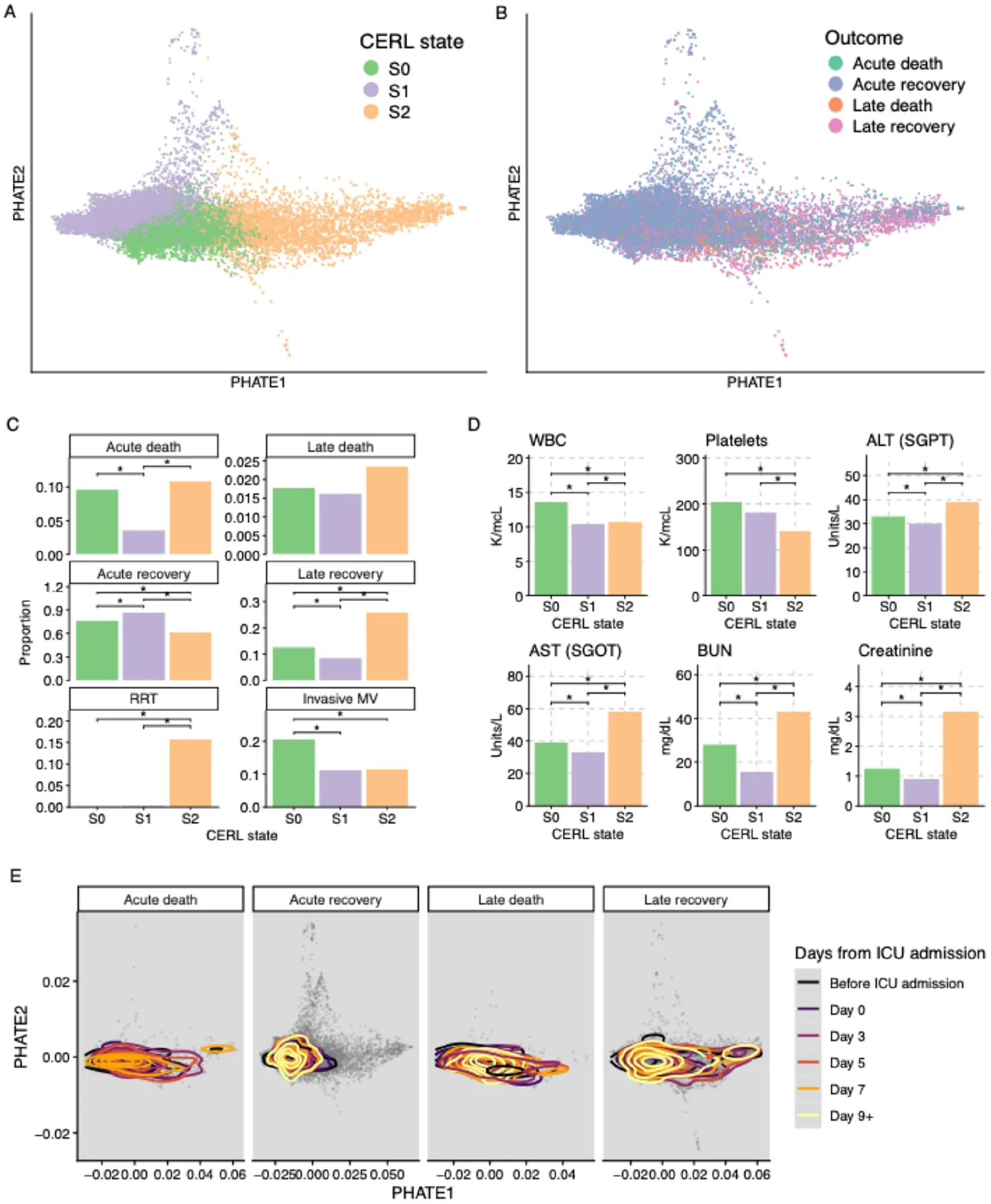
Real-world application of CERL to longitudinal sepsis data. CERL was applied to patients with sepsis from the eICU Collaborative Research Database (eICU-CRD) to characterize continuous and discrete biological and clinical state organization. A) Projection of the continuous CERL representation using Potential of Heat-diffusion for Affinity-based Transition Embedding (PHATE), with discrete CERL states (S0, S1, and S2) derived by K-means clustering of the CERL representations. B) Four clinical trajectories defined using longitudinal Sequential Organ Failure Assessment (SOFA) scores and used as clinical trajectory context for CERL representation learning. C) State-specific proportions of prespecified clinical trajectories among observations at ICU admission (day 0). Pairwise differences of clinical trajectory proportion between CERL states were assessed using Fisher’s exact tests with Benjamini–Hochberg correction for multiple comparisons. An asterisk () indicates an adjusted P < 0.05. D) Selected laboratory measurements across CERL states. Laboratory measurements obtained from one day before to one day after ICU admission were summarized by the mean value for each measurement. Differences across CERL states were evaluated using linear mixed-effects models with patient-specific random intercepts to account for repeated measurements. Pairwise comparisons were performed with Tukey adjustment for multiple comparisons; an asterisk () indicates an adjusted P < 0.05. E) Temporal density redistribution of observations associated with each clinical trajectory across the continuous CERL-derived PHATE representation, illustrating changes in population-level state distributions over time.

## Data Availability

This study did not generate new materials.

## Data and code availability

Data used in this study are available through PhysioNet to credentialed users who complete the required training and data use agreement. R and python scripts used for data preprocessing and analysis are available from https://github.com/Kamaleswaran-Lab/cerl.
